# Plasticity of intrinsic excitability across the estrous cycle in hypothalamic CRH neurons

**DOI:** 10.1038/s41598-021-96341-4

**Published:** 2021-08-17

**Authors:** Emmet M. Power, Karl J. Iremonger

**Affiliations:** Centre for Neuroendocrinology, Department of Physiology, School of Biomedical Sciences, University of Otago, Dunedin, 9016 New Zealand

**Keywords:** Cell biology, Neuroscience, Physiology, Endocrinology

## Abstract

Stress responses are highly plastic and vary across physiological states. The female estrous cycle is associated with a number of physiological changes including changes in stress responses, however, the mechanisms driving these changes are poorly understood. Corticotropin-releasing hormone (CRH) neurons are the primary neural population controlling the hypothalamic–pituitary–adrenal (HPA) axis and stress-evoked corticosterone secretion. Here we show that CRH neuron intrinsic excitability is regulated over the estrous cycle with a peak in proestrus and a nadir in estrus. Fast inactivating voltage-gated potassium channel (I_A_) currents showed the opposite relationship, with current density being lowest in proestrus compared to other cycle stages. Blocking I_A_ currents equalized excitability across cycle stages revealing a role for I_A_ in mediating plasticity in stress circuit function over the female estrous cycle.

## Introduction

The female estrous cycle is associated with multiple changes in physiology and behaviour, including marked changes in stress responses^1,2^. The hypothalamic–pituitary–adrenal (HPA) axis is most active during the proestrus stage of the estrous cycle. Basal circulating levels of corticosterone (CORT) are highest on proestrus^3–6^, as are stress-evoked secretion of both CORT and ACTH^7^. There are also notable changes in stress and anxiety associated behaviours over the estrous cycle^1,2,8–10^. The mechanisms which drive these estrus cycle dependent changes in stress responses are currently unclear.

Hypothalamic corticotropin-releasing hormone (CRH) neurons control both the HPA axis and behavioral responses to stress^11–17^. CRH neuron excitability and stress responses are highly plastic, thus allowing organisms to mount appropriate stress responses in different behavioral or physiological states. Adaptation of CRH neuron responses is thought to be mediated by synaptic plasticity^18^, however, recent evidence suggests that plasticity of intrinsic excitability also plays an important role^19–21^. Plasticity of intrinsic excitability is mediated by changing levels of expression or function of ion channels in the cell membrane^22–24^. In CRH neurons, plasticity of intrinsic excitability can be mediated via voltage-gated potassium channels^21^. In other neurons, expression of voltage-gated potassium channels has also been shown to be regulated over the estrous cycle^25,26^. Hence, we speculated that changes to potassium channels influences intrinsic excitability of CRH neurons over the estrous cycle.

## Results

### CRH neuron intrinsic excitability changes over the estrous cycle

**Figure 1:**
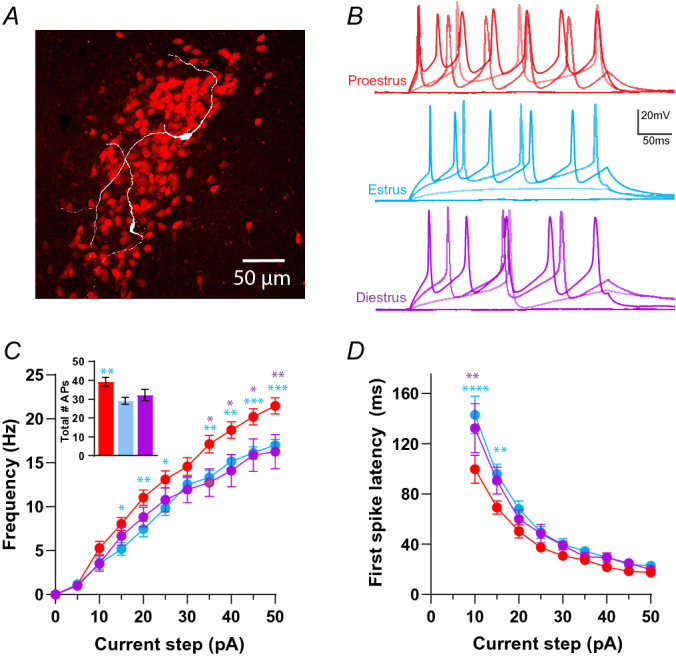
CRH neuron intrinsic excitability varies across the female estrous cycle. (**A**) Image of left hemisphere PVN showing expression of tdTomato (red) in CRH neurons. White cells are tdTomato positive CRH neurons that have been filled with neurobiotin via the whole cell recording pipette. (**B**) Representative responses of CRH neurons to 0 pA, 10 pA, 30 pA and 50 pA current steps. Proestrus in red, estrus in blue and diestrus in purple. (**C**) Summary data for the F/I curve. Cells from proestrus animals (n = 29 cells) show a significantly higher firing frequency compared to those from estrus (n = 38) and diestrus (n = 22). Symbols denote significance determined by Tukey’s post hoc multiple comparisons test (Two-way ANOVA results in Supplementary Table 1), blue: proestrus versus estrus, purple: proestrus versus diestrus. Insert shows total number of APs fired over all current steps for each group. (**D**) Graph of first spike latency (FSL) for each current step. Cells from proestrus animals had a shorter FSL at 10 and 15 pA steps. Stars are results of post hoc multiple comparisons, as in B. N = 9, 15 and 10 mice for diestrus, estrus, and proestrus groups respectively. *P* values: * ≤ 0.05, ** ≤ 0.01, *** ≤ 0.001, **** ≤ 0.0001.

To assay CRH neuron intrinsic excitability, neurons were held around − 60 mV in current clamp before injecting a family of current steps from 0 pA to + 50 pA in 5 pA increments (Fig. 1C). This protocol was performed on CRH neurons from female animals in proestrus, estrus or diestrus stages of the estrous cycle. A Two-way RM ANOVA revealed that there was a significant effect of estrous cycle stage on CRH neuron spiking responses (F_(2,86)_ = 4.387, *P* = 0.0153, Fig. 1C, Table 1), a significant effect of current step (F_(10,860)_ = 470, *P* < 0.0001, supplementary Table 1) and a significant interaction (F_(20,860)_ = 3.158, *P* < 0.0001, supplementary Table 1). Post hoc tests showed that at multiple current steps excitability in proestrus was significantly higher than both estrus (*P* < 0.05) and diestrus (*P* < 0.05, Fig. 1B). Peak firing frequency (frequency at the 50 pA step) of CRH neurons from proestrus animals was significantly higher than estrus and diestrus (F_(2,86)_ = 6.62. *P* = 0.002, Fig. 1C insert). Additionally, the slopes of the F/I curves for each group were significantly different (F_(2,79)_ = 5.49, *P* = 0.006) (see Table 1 for values and Supplementary Table 1 for full statistics), with the highest gain in proestrus compared to estrus (*P* = 0.0044). The slope of the F/I curve was not significantly different between proestrus and diestrus (*P* = 0.1493). The total number of action potentials (APs) fired over all current steps was also significantly different (one-way ANOVA, F_(2,83 )_ = 5.35, *P* = 0.006; Table 1) with post hoc tests showing higher numbers of spikes in proestrus compared to the estrus group (*P* = 0.005). Interestingly, analysis of the AP parameters showed no differences between the groups. AP amplitude, rise time, half width and decay time were not significantly different (Table 1). There were also no significant differences in capacitance or input resistance between groups (one-way ANOVA, F_(2,104)_, *P* = 0.59 and F_(2,101)_ = 1.05, *P* = 0.35 respectively, Table 1). We next measured first spike latency (FSL) to determine if this was different across the estrous cycle. A Two-way RM ANOVA showed that there was a significant effect of estrous cycle stage on FSL (F_(2,47)_ = 3.57, *P* = 0.036), a significant effect of current step (F_(8,376)_ = 143.2, *P* < 0.0001) and a significant interaction (F_(16,376)_ = 2.16, *P* = 0.006). Post hoc tests revealed that CRH neurons from proestrus animals exhibited a significantly shorter latency to fire an action potential (AP) at the 10 and 15 pA current steps compared to estrus (*P* < 0.05) and diestrus groups (*P* < 0.05, Fig. 1D).

**Table 1:** Electrophysiological properties of CRH neurons at different stages of the estrous cycle.

Name	Group	Mean value ± SEM
Total # APs fired during F/I curve	Proestrus	39.24 ± 2.35
	Estrus	29.22 ± 1.85**
	Diestrus	32.27 ± 2.97
Slope of F/I curve	Proestrus	0.446 ± 0.018 Hz/pA
	Estrus	0.366 ± 0.014 Hz/pA**
	Diestrus	0.386 ± 0.034 Hz/pA
Peak firing rate (50pA step)	Proestrus	22.27 ± 0.93 Hz
	Estrus	16.65 ± 0.66 Hz**
	Diestrus	16.26 ± 1.93 Hz*
AP amplitude	Proestrus	85.6 ± 3.7 pA
	Estrus	83.16 ± 1.25 pA
	Diestrus	81.82 ± 1.68 pA
AP rise time	Proestrus	0.37 ± 0.03 ms
	Estrus	0.35 ± 0.016 ms
	Diestrus	0.34 ± 0.025 ms
AP half width	Proestrus	1.29 ± 0.12 ms
	Estrus	1.28 ± 0.08 ms
	Diestrus	1.16 ± 0.12 ms
AP decay time	Proestrus	0.93 ± 0.1 ms
	Estrus	0.82 ± 0.04 ms
	Diestrus	0.82 ± 0.1 ms
Input resistance	Proestrus	1.79 ± 0.185 GΩ
	Estrus	1.52 ± 0.117 GΩ
	Diestrus	1.59 ± 0.104 GΩ
Capacitance	Proestrus	19.17 ± 1.367 pF
	Estrus	18.54 ± 0.866 pF
	Diestrus	17.53 ± 1.05 pF

Mean values for each group (proestrus, estrus and diestrus) for the listed properties. Stars indicate statistical significance with the proestrus group (full statistical results given in Supplementary Table 1). AP stands for action potential. *P* values: * ≤ 0.05, ** ≤ 0.01.

Taken together these data show that CRH neuron excitability fluctuates over the estrous cycle with higher levels of excitability during proestrus compared to estrus and diestrus stages. These findings align well with previous reports showing higher levels of HPA axis activity in proestrus^7,27^.

### Changes in I_A_ potassium current across the estrous cycle

**Figure 2:**
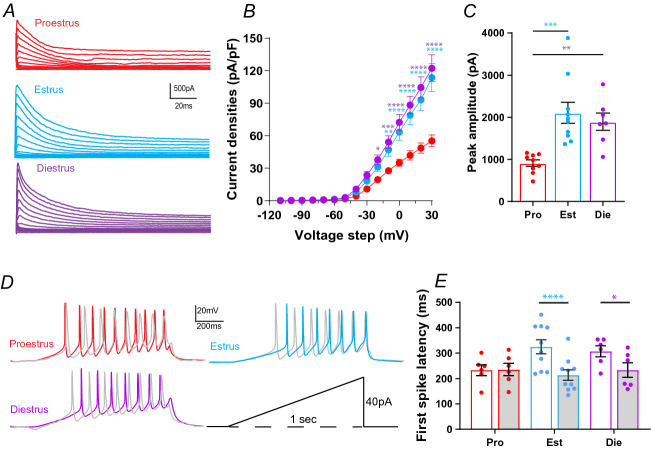
I_A_ currents vary across the estrous cycle influencing FSL. (**A**) Example I_A_ currents evoked from individual cells during each cycle stage; proestrus (red), estrus (blue) and diestrus (purple). (**B**) I_A_ current densities plotted for each 10 mV voltage step. Cells from proestrus (n = 8) animals had significantly smaller I_A_ currents compared to those from estrus (n = 9) and diestrus (n = 7) animals. (**C**) Peak amplitude I_A_ currents (not normalized to capacitance) evoked by a + 30 mV step. Peak I_A_ currents during proestrus were significantly smaller than during other cycle stages. (**D**) Example traces showing CRH neuron spiking response to a one second current ramp protocol (bottom right). Grey traces are example traces from each group in the presence of 4-AP. (**E**) Bar graph of first spike latency, measured from the onset of the ramp to AP threshold, for each group under control conditions and in the presence of 4-AP (grey bars). 4-AP had a significant effect on FSL in cells from estrus and diestrus animals but did not affect FSL in proestrus animals. Results of one and two-way ANOVAs reported in Supplementary Table 1. Star symbols denote significance by Tukey’s multiple comparisons test, purple: diestrus versus proestrus, blue: estrus versus proestrus. There were no significant differences between estrus and diestrus groups. N = 5–8 mice for all groups. *P* values: * ≤ 0.05, ** ≤ 0.01, *** ≤ 0.001, **** ≤ 0.0001.

Changes in intrinsic excitability are commonly due to changes in voltage-gated ion channel density or function. I_A_ currents contribute to FSL in CRH neurons^21^ and expression of potassium channel subunits which contribute to I_A_ current change over the estrous cycle in other neurons^25^. As FSL was found to vary across the estrous cycle we subsequently used a voltage clamp protocol (see “Methods”) to determine if I_A_ currents exhibited similar changes. A Two-way RM ANOVA revealed that there was a significant effect of estrous cycle on I_A_ current density (F_(2,21)_ = 9.59, *P* = 0.001; Fig. 2A,B), a significant effect of voltage step (F_(14,294)_ = 239.8, *P* < 0.0001) and a significant interaction (F_(28,295)_ = 10.5, *P* < 0.0001). Post hoc tests showed that current densities at multiple voltage steps were lowest in proestrus animals compared to estrus (*P* < 0.05) and diestrus animals (*P* < 0.05). Peak amplitude of the current at the maximum voltage step (+ 30 mV) was also significantly different between groups (One-way ANOVA, F_(2, 23)_ = 10.93, *P* = 0.0005, Fig. 2C). There was no significant difference in the decay tau of the I_A_ currents between the groups (Tau measured at + 30 mV step, One-way ANOVA, F_(2,30)_ = 0.51, *P* = 0.603, data not shown). Voltage dependence of inactivation and time course of recovery from inactivation were also not significantly different between the groups (Two-way ANOVA, F_(2,8)_ = 1.92, *P* = 0.201, and F_(2,7)_ = 0.44, *P* = 0.66, respectively; data not shown).

### Role of I_A_ in estrous cycle changes in CRH neuron excitability

To determine if changes in I_A_ currents contributed to the differences in intrinsic excitability observed, we measured FSL with a ramp protocol (0 to 40 pA ramp over one second, Fig. 2D). We then applied 2 mM 4-AP to inhibit I_A_ currents and repeated the ramp protocol. Two-way ANOVA revealed a significant effect of 4-AP on FSL (F_(1,19)_ = 19.2, *P* = 0.0003, Fig. 2E), no significant effect of estrous cycle stage (F_(2,19)_ = 0.7432, *P* = 0.4889), but a significant interaction between estrous cycle stage and 4-AP (F_(2,19)_ = 6.06, *P* = 0.009). While FSL was significantly shorter in the proestrus group compared to the estrus group in control (*P* = 0.039), there was no significant difference between the groups in the presence of 4-AP. Consistent with this, 4-AP significantly reduced FSL in the estrus (*P* < 0.0001) and diestrus (*P* = 0.03) groups, but not in the proestrus group (*P* = 0.999, Fig. 2D,E).

## Discussion

There are marked changes in activity of the HPA axis across the female reproductive cycle. Basal circulating levels of CORT are highest on proestrus^3–6^, as are stress evoked secretion of both CORT and ACTH^7^. Here we show that CRH neuron intrinsic excitability is also regulated across the estrous cycle with a similar pattern. Specifically, CRH neuron spiking excitability and gain were found to be highest in proestrus and lowest in estrous. FSL and I_A_ current density were lowest in proestrus. Differences in FSL across estrous cycle stages were abolished when I_A_ currents were inhibited with 4-AP.

Expression of voltage-gated potassium channel subunits have previously been shown to vary over the estrous cycle in GnRH neurons^25^. In these same neurons, estradiol can also regulate voltage-gated potassium channel function^28^. It is tempting to suggest that the increases in CRH neuron excitability at proestrus are also mediated directly by estradiol since estradiol levels peak at this stage of the estrous cycle^29^. However, several studies have shown that when delivered in vivo to ovariectomized rats, estradiol suppresses stress evoked CRH neuron activity assessed by cfos^30–32^. Exogenous estradiol can however, enhance corticosterone secretion via direct actions on the adrenal gland^30,33,34^. Therefore, it is worth considering that when estradiol levels are artificially elevated in ovariectomized animals, changes in CRH neuron excitability may also be influenced by changing levels of corticosterone negative feedback. It is also important to note that CRH neurons do not appear to possess estrogen receptor (ER) alpha^35^ and have little ERbeta^36^. This has led to the idea that any estradiol actions on CRH neurons may either be mediated by membrane associated estrogen receptors^37^ or be mediated indirectly through the regulation of afferent neural circuits^31,38,39^. Since progesterone levels also vary over the estrous cycle, it too may play a role in regulating CRH neuron excitability. While the role of estradiol and progesterone could be addressed by giving steroid implants in ovariectomized animals, these experiments do not mimic the natural fluctuations in these hormones and may therefore lead to erroneous conclusions as to their normal effects on CRH neuron function.

Overall, our findings demonstrate plasticity of CRH neuron intrinsic excitability over the course of the natural estrous cycle which may in turn underlie changes in HPA axis output. In addition to controlling adrenal corticosteroid secretion, CRH neurons also play an important role in regulating stress associated behaviours^14,15,17,40^. Hence, changes in CRH neuron excitability may also contribute to estrous cycle changes in behavioural stress responses.

## Methods

### Animals

All experiments were carried out in adult female (2–6 months old) Crh-IRES-Cre;Ai14 (tdTomato) mice. These mice faithfully label CRH neurons in the PVN (Fig. 1A)^41–43^. Animals had a 12 h light/dark cycle (7 a.m.–7 p.m. lights on) with food and water available ad libitum. A small amount of male bedding was put in the cages each week to promote normal estrous cycling. To establish estrous cycle stage, animals were vaginally smeared post mortem^44^. Animals in metestrus and diestrus were combined into one group and referred to as diestrus. All protocols and procedures were approved by the University of Otago Animal Ethics Committee and carried out in accordance with the New Zealand Animal Welfare Act. The study was carried out in compliance with the ARRIVE guidelines.

### Slice preparation

Mice were killed by cervical dislocation between 9 and 11 a.m., their brain quickly removed and placed in ice-cold oxygenated (95% O_2_, 5% CO_2_) slicing solution containing (in mM); 87 NaCl, 2.5 KCl, 25 NaHCO_3_, 1.25 NaH_2_PO_4_, 0.5 CaCl_2_, 6 MgCl_2_, 25 d-Glucose, 75 sucrose, pH 7.2–7.4. A vibratome (VT1200S, Lecia Microsystems) was used to cut 200 µm-thick coronal slices of the PVN, which were then incubated in oxygenated artificial cerebrospinal fluid (aCSF) containing in (mM); 126 NaCl, 2.5 KCl, 26 NaHCO_3_, 1.25 NaH_2_PO_4_, 2.5 CaCl_2_, 1.5 MgCl_2_, 10 d-Glucose at 30 °C for at least 1 h before recording. For recording, slices were transferred to a recording chamber and continuously perfused with 30 °C aCSF at 1.5 ml min^−1^. CRH neurons within the PVN were visualized using a 40× objective and epifluorescence to excite tdTomato.

### Whole-cell electrophysiology recordings

Electrophysiological recordings were collected with a Multiclamp 700B amplifier (Molecular Devices), filtered at 2 kHz, and digitized using the Digidata 1440a (Molecular Devices). Data were analysed with Clampfit 10.7 (Molecular Devices).

For whole-cell recordings, borosilicate glass pipettes (tip resistance: 2–5 MΩ) were filled with an internal solution containing (in mM): 120 K‐gluconate, 15 KCl, 0.5 Na_2_EGTA, 2 Mg_2_ATP, 0.4 Na_2_GTP, 10 HEPES, 5 Na_2_‐phosphocreatine and 0.25% Neurobiotin (adjusted to pH 7.2 with KOH; adjusted to ≈ 290 mOsm with sucrose). All current clamp experiments were performed in the presence of 10 μM cyanquixaline (6-cyano-7-nitroquinoxaline-2,3-dione) (CNQX) and picrotoxin (50 μM). Each cell was held at approximately − 60 mV. The liquid junction potential was calculated to be approximately − 14.1 mV and was not compensated for. Cells were not recorded from if input resistance was below 0.7 GΩ or access resistance was above 30 MΩ and both input and access resistance were monitored throughout to ensure stable recording. We used a current step protocol to determine spike output and first spike latency (FSL) for Fig. 1. The step protocol consisted of 300 ms square steps from 0 to + 50 pA in 5 pA increments. Spikes were detected using a threshold search in Clampfit and were analysed for rise time, decay time, amplitude and half width. FSL was calculated from the time of the depolarizing step initiation to the action potential (AP) threshold for the first spike evoked at steps equal or greater than 10 pA. AP threshold was defined as the voltage at which the AP first derivative crossed 10 mV/ms. The same analysis criteria were used to identify FSL and AP threshold for a 1 s, + 40 pA/s ramp protocol (Fig. 2). Slopes of the F-I curve for individual cells were calculated using linear regression.

For all voltage clamp recordings neurons were clamped at − 60 mV, input resistance, access resistance and capacitance were monitored periodically throughout recordings. I_A_ current recordings were performed in the presence of CNQX (10 μM), picrotoxin (50 μM), TTX (0.5 μM), TEA (30 mM) (or XE991, 40 μM) and nifedipine (100 μM). To evoke I_A_ currents, neurons were hyperpolarized from − 60 to − 110 mV for 500 ms before a family of depolarizing steps were delivered in 10 mV steps from − 100 to + 30 mV. Peak I_A_ amplitude for each voltage step was measured and normalized to capacitance to give the current densities (pA/pF). Voltage dependence of inactivation and time course of recovery from inactivation protocols are the same as used in Sonner and Stern^45^. Four mM 4-Aminopyridine (4-AP) blocked peak I_A_ current amplitude by 90.7 ± 3.83% (n = 3).

### Immunohistochemistry and confocal microscopy

Slices containing neurobiotin filled cells were fixed with 4% paraformaldehyde in phosphate buffered solution overnight at 4 °C. Similar to the protocol reported in^46^, slices then underwent immunohistochemical staining using Cy5-Streptavidin secondary antibody (1:30 dilution in TBS, Invitrogen). Slices were mounted on slides, air dried and coverslipped using mounting media (Vectorshield, Vector Laboratories).

Confocal images were then acquired on a Nikon A1R confocal microscope with a 10× objective. tdTomato was excited with a 560 nm laser and Cy5 was excited with a 635 nm laser, images were acquired at 1024 × 1024 pixels.

### Analysis

Statistical analysis was performed using GraphPad Prism 8. All reported values are the mean ± SEM. Comparisons between groups were carried out using either One or Two-way ANOVA where appropriate, with Tukeys or sidak’s post hoc multiple comparison tests. All n-values represent neuron number, all groups had N > 3 animals. *P* < 0.05 was considered statistically significant. Full ANOVA results are reported in Supplementary Table 1. P values reported on figures are for post hoc multiple comparison tests.

## Supplementary Information

Supplementary Information.
