# Supplementary material for: Plasticity of intrinsic excitability across the estrous cycle in hypothalamic CRH neurons
Source: Sci Rep. 2021 Aug 17;11:16700. doi: 10.1038/s41598-021-96341-4 (PMC8371084; doi:10.1038/s41598-021-96341-4)
Supplement: Supplementary file 1 — Supplementary Information. [file 41598_2021_96341_MOESM1_ESM.docx]

*Supplementary Table 1:*

| **Name** | **Fig#** | **Statistical test** | **ANOVA table** | **F (DFn, DFd)** | ***P* value** |
| --- | --- | --- | --- | --- | --- |
| F/I | 1.B | RM two-way ANOVA | Current step x Cycle  Current step  Estrous cycle | (20, 860)=3.158  (10, 860)=470  (2, 86) = 4.387 | <0.0001  <0.0001  0.0153 |
| Slope of F/I curve | - | One-way ANOVA | Estrous cycle | (2, 79)=5.49 | 0.006 |
| FSL | 1.C | RM two-way ANOVA | Current step x Cycle  Current step  Estrous cycle | (16, 376)=2.16  (8, 376)=143.2  (2, 47)=3.57 | 0.006  <0.0001  0.036 |
| F/I total# of APs | - | One-way ANOVA | Estrous cycle | (2, 83)=5.35 | 0.0065 |
| AP amplitude | - | One-way ANOVA | Estrous cycle | (2, 69)=0.634 | 0.533 |
| AP rise time | - | One-way ANOVA | Estrous cycle | (2, 66)=0.432 | 0.651 |
| AP half width | - | One-way ANOVA | Estrous cycle | (2, 70)=0.47 | 0.627 |
| AP decay time | - | One-way ANOVA | Estrous cycle | (2, 69)=0.557 | 0.589 |
| I_A_ current densities | 2.B | RM two-way ANOVA | Voltage step x Cycle  Voltage step  Estrous cycle | (28, 294)=10.5  (14, 294)=239.8  (2, 21)=9.59 | <0.0001  <0.0001  0.001 |
| I_A_ peak amplitude | 2.C | One-way ANOVA | Estrous cycle | (2, 23)=10.93 | 0.0005 |
| Effect of 4-AP on FSL | 3.B | RM two-way ANOVA | 4-AP x Cycle  4-AP  Estrous cycle | F_(2,19)_=6.06  F_(1,19)_=19.18  F_(2,19)_=0.74 | 0.009  0.0003  0.489 |

**Supplementary Table 1:**

Table reporting detailed statistical results from one and two-way ANOVAs reported in the text. Multiple comparison results are reported on figures and in figure legends.
